# Analgesic Effect of Sulforaphane: A New Application for Poloxamer-Hyaluronic Acid Hydrogels

**DOI:** 10.3390/gels10070460

**Published:** 2024-07-13

**Authors:** Juliana Zampoli Boava Papini, Bruno de Assis Esteves, Vagner Gomes de Souza Oliveira, Henrique Ballassani Abdalla, Cintia Maria Saia Cereda, Daniele Ribeiro de Araújo, Giovana Radomille Tofoli

**Affiliations:** 1 Faculdade São Leopoldo Mandic, Instituto de Pesquisa São Leopoldo Mandic, Rua José Rocha Junqueira 13, Campinas 13045-75, SP, Brazil; 2 Centro de Ciências Naturais e Humanas, Universidade Federal do ABC, Av. dos Estados, 5001. Bloco A, Torre 3, Santo André 09210-580, SP, Brazil; daniele.araujo@ufabc.edu.br; 3 Escola Paulista de Medicina, Departamento de Biofísica, Universidade Federal de São Paulo, Rua Botucatu, 862, Vila Clementino, Sao Paulo 04023-062, SP, Brazil

**Keywords:** sulforaphane, poloxamer, postoperative pain, hyaluronic acid, drug delivery

## Abstract

Sulforaphane (SFN) has shown potential as an antioxidant and anti-inflammatory agent. To improve its druggability, we developed new analgesic formulations with sulforaphane-loaded hyaluronic acid (HA)-poloxamer (PL) hydrogel. This study evaluated the pre-clinical safety and effectiveness of these formulations. Effectiveness was tested on Wistar rats divided into groups (n = 15) receiving (IM, 10 mg/kg) SFN formulations or control groups (without SFN). This study used a hind paw incision postoperative pain model to evaluate mechanical hypersensitivity with von Frey filaments. TNF-α, IL-1β, substance P, and CGRP levels verified anti-inflammatory activity in the hind paw tissue. Histopathology of tissues surrounding the injection site was assessed after 2 and 7 days post-treatment. To corroborate drug safety, cell viability of 3T3 and RAW 264.7 cultures was assessed. Additionally, RAW 264.7 cultures primed with carrageenan evaluated nitric oxide (NO) levels. All animals exhibited post-incisional hypersensitivity, and F2 (PL 407/338 (18/2%) + HA 1% + SFN 0.1%) showed a longer analgesic effect (*p* < 0.05). F2 reduced TNF-α, IL-1β, and CGRP levels (*p* < 0.05). Histopathological evaluation showed mild to moderate inflammatory reactions after the formulations’ injections. F2 produced no significant difference in cell viability (*p* > 0.05) but reduced NO production (*p* < 0.05). Thus, our results highlight the biocompatibility and effectiveness of F2.

## 1. Introduction

Postoperative pain is usually treated with opioids, local anesthetics, and non-steroidal anti-inflammatory drugs (NSAIDs) [1,2]. However, opioids can cause serious adverse effects such as respiratory depression, constipation, tolerance, and chemical dependence [3]. Opioid dependence is responsible for numerous substance use disorders and deaths, with nearly 80% of patients with this disorder remaining undiagnosed. Overdose deaths involving opioids in the USA rose from 21,088 in 2010 to 49,860 in 2019 [4]. Additionally, opioids mainly act within the central nervous system to provide analgesia, without addressing the inflammatory component of pain. Addressing the inflammatory response may reduce the need for opioids and improve recovery after surgery [5].

Local anesthetics, commonly administered by injection at the surgical site, may have a short duration and adverse systemic effects [1,6]. NSAIDs also have significant adverse effects, such as platelet dysfunction, gastrointestinal irritation, and renal dysfunction caused by COX inhibition [7].

In this context, there is a growing need for non-opioid alternatives to control postoperative pain. Therefore, we propose using new formulations of sulforaphane (SFN) in hydrogels to promote postoperative pain control. SFN is a compound abundantly found in the seeds and sprouts of cruciferous plants, such as broccoli, cauliflower, brussels sprouts, cabbage, kale, watercress, and bok choy [1]. SFN is present in these plants in the form of its chemical precursor, glucoraphanin. When plants are cut or chewed, this substance is hydrolyzed into SFN either by the plant enzyme myrosinase or by bacterial thioglucosidase in the colon [8].

SFN induces the Nrf2 pathway and inhibits NF-κB, thus activating antioxidant and anti-inflammatory responses [9]. Previous reports show that SFN presents anti-diabetic/anti-hyperlipidemic, antiangiogenic, antioxidant, neuroprotective, anti-aging, cardioprotective, anti-inflammatory, and antimicrobial activities. SFN also shows activity against different types of cancer and has been effective against various neurodegenerative diseases [10].

SFN’s properties make it a promising candidate for postoperative pain treatment. This paper reports on an innovative use of hyaluronic acid and poloxamer (HA/PL) hydrogel systems for SFN delivery. It aims to evaluate the use of this in postoperative pain treatment through intramuscular injections. This investigation uses new SFN formulations initially designed to be administered through intra-articular injections to alleviate osteoarthritis symptoms and prevent disease progression. Our research group recently described these formulations in terms of their physicochemical characteristics, biopharmaceutical effects, and pharmacological efficacy in in-vitro and ex-vivo osteoarthritis models [1]. We reported the hydrogel supramolecular structure, sol-gel transition, and mechanical properties (by using different techniques such as SAXS, SANS, rheology, FTIR, DSC, DLS, and SLS) as well as their relationships with release constants and mathematical models. However, there was a claim for more detailed investigations on pharmacological fields. In this sense, this article intended to demonstrate the main pharmacological properties, compressing the analgesic effects (effectivity, molecular mechanisms, and biocompatibility) of poloxamer-based hydrogels developed by our group. This study’s data from our previous work support that SFN has several advantages when used in the form of an injectable hydrogel for in situ gelling, such as simple preparation and physicochemical stability. Additionally, the HA/PL formulations were structured as liquid-viscous gels in which viscoelastic behavior predominates. This is an essential property for developing adequate injectable hydrogel formulations.

Besides presenting pharmaceutical advantages, the three main components of the formulation tested in this study are considered safe and non-toxic. SFN’s safety and efficacy in humans have been demonstrated by several clinical studies [10]. HA presents a high potential for drug loading and is both biodegradable and biocompatible. Furthermore, HA can serve as a targeting ligand at inflammation sites due to its specific recognition of stabilin-2 and CD44 receptors, which are present in high-affinity forms on activated macrophages and on the surfaces of T and B lymphocytes [11]. Finally, PL’s principal asset is its biocompatibility derived from its capacity for forming gels close to body temperature. There are no organic solvent requirements or pH variations for these substances’ hydrogel preparations [12].

Despite these conveniences, the above-described formulations have not yet been tested in a pain state presenting inflammation and hyperalgesia. Thus, this study proposes to evaluate a new strategy for postoperative pain control in animal models. We assess if HA/PL hydrogel formulations can provide the sustained release and prolonged analgesia essential for effective in-vivo pain control. Additionally, we evaluate the system’s effect in a RAW-cell culture model. The investigation also analyzes local toxicity after drug application through histological assessment of the injection site and 3T3 fibroblast cell cultures. Our results showed that the combination of poloxamer 407/338 (18/2%), hyaluronic acid (1%), and sulforaphane (0.1%) presents biocompatibility and effectiveness for pain control. This new formulation enhances the analgesic effect of sulforaphane and reduces TNF-α, IL-1β, and CGRP levels after the induction of postoperative pain.

## 2. Results and Discussion

Many animal models have been established to mimic human disease conditions in preclinical trials. In this study, we used a postoperative animal pain model to mimic inflammatory pain and propose a new kind of treatment for this condition. Two main reasons stimulated our group’s pursuit of a new analgesic tool.

The first is that postoperative pain management is a persistent healthcare challenge. Currently, there are pressing needs to accelerate recovery, increase patient satisfaction, reduce the length of postoperative hospital stays, and decrease the number of opioid prescriptions [13]. Relatedly, the second reason is that the growing opioid epidemic in the United States has increased our understanding of these drugs’ detrimental effects on society [14]. According to the literature, this phenomenon has had various impacts, affecting both the medical sphere (in terms of prescription numbers, clinical consequences, and comorbidities) and the social and financial spheres, which involve patients, the general population, and the healthcare system [15]. In this context, there is a growing need for non-opiate alternatives to promote postoperative pain control, such as our new SFN formulations.

Throughout the development of this new strategy for postoperative pain management, we used new SFN formulations that were initially designed for intra-articular route application and osteoarthritis treatment. Thus, our study aimed to verify if these formulations would be safe and effective for treating inflammatory postoperative pain when applied through intramuscular injections. For this purpose, we used both in-vitro and in-vivo models.

**Figure 1:**
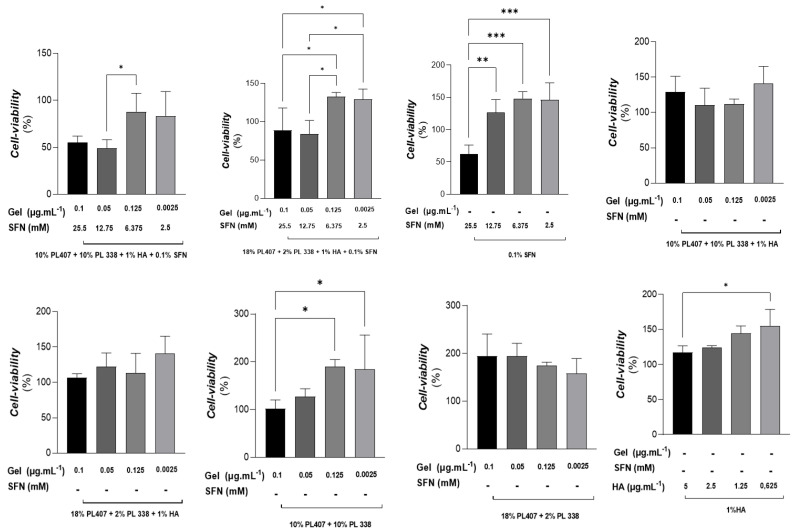
Cell viability at 570 nm (mean ± SD, n = 4) for the MTT assay on Balb/c 3T3 fibroblast cells after 24 h of treatment with SFN formulations (F1, F2, and F3) and control group formulations (without SFN—C1 to C5). One-way analysis of variance with Tukey post-hoc test: *** *p* < 0.001; ** *p* < 0.01; * *p* < 0.05. The intragroup analysis is represented in the figure with brackets. Intergroup analysis: Concentration 2.5 mM SFN; 0.0125 µg·mL^−1^ PL and HA 0.625 mg·mL^−1^: F1 < F2 * and F3 ***. F1 < C1, C3, C4 * and C5 **. Concentration 6.375 mM SFN; 0.025 µg·mL^−1^ PL and HA 1.25 mg·mL^−1^: F1 < F2, F3, C1, C2, C3, C4, C5; F2 < C3 ** and C4 ***; F3 > C1 and C2 *; F3 > F6 **; C1 < C3 and C4; C2 < C3 and C4. Concentration 12.75 mM SFN; 0.05 µg·mL^−1^ PL and HA 2.5 mg·mL^−1^: C4 > F1 and C2; C4 > F3, C3, C5 **; C4 > C1 and C2 ***. Concentration 25.5 mM SFN; 0.1 µg·mL^−1^ PL and HA 5 mg·mL^−1^: F1 < C1 **; C4 and C5 *; F3 < C1** and C4; C4 > C1 **; C2 ***; C3 *** and C5 **.

To assess the possible local toxicity of the formulations, we evaluated cell viability using two cell types. In the first assay, we used Balb/c 3T3 fibroblasts treated with four different concentrations of the SFN formulations (F1 to F3) or the control groups (C1 to C5) (Figure 1). In the intergroup analysis, we found that, after 24 h, F1 in all concentrations decreased cell viability when compared to the four control group formulations that did not contain SFN (*p* < 0.05). Additionally, we observed that, even in the formulations with the two lower concentrations (6.375 and 2.5 mM of SFN), F1 still decreased cell viability when compared to the control groups and F2 (*p* < 0.05). However, we found that F2 only reduced cell viability when compared to the control groups receiving C3 and C4 (*p* < 0.05). In the intragroup analysis, we generally observed that higher concentrations of SFN affected cell viability (*p* < 0.05), while the lower concentrations did not present this effect.

**Figure 2:**
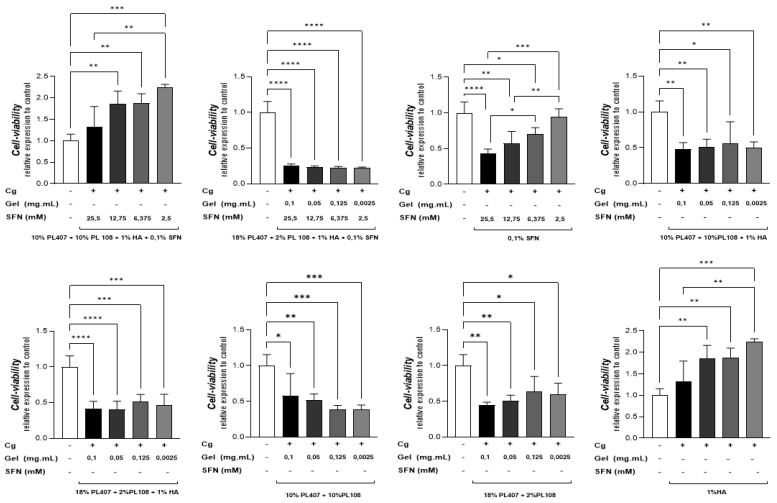
Cell viability at 570 nm (mean ± SD, n = 4) on RAW 264.7 macrophages after 24 h of treatment with SFN formulations (F1, F2, and F3) and control groups (without SFN—C1 to C5). One-way analysis of variance with Tukey post-hoc test: **** *p* < 0.0001; *** *p* < 0.001; ** *p* < 0.01; * *p* < 0.05. The intragroup analysis is represented in the figure with brackets. Intergroup analysis: Concentration 2.5 mM SFN; 0.0125 µg·mL^−1^ PL and HA 0.625 mg·mL^−1^: F1 < F3 ***, C1 *, C4 ***; F2 < F3 ****, C1 ** and C5 ***; F3 > C1, C2 and C4 ***; C5 > F1, F2, F3, C1, C2, C3 and C4 ****. Concentration 6.375 mM SFN; 0.025 µg·mL^−1^ PL and HA 1.25 mg·mL^−1^: F1 < F3 **, C1 *, C4 **; F2 < F3 **, C1 and C4 **; C5 > F1, F2, F3, C1, C2, C3 and C4 ****. Concentration 12.75 mM SFN; 0.05 µg·mL^−1^ PL and HA 2.5 mg·mL-1: F1 < F3 **; F1 < C1,C3, C4 *; F2 < F3, C4 **; F2 < C3 and C4 *; C5 > F1, F2, F3, C1, C2, C3 and C5 ****. Concentration 25.5 mM SFN; 0.1 µg·mL^−1^ PL and HA 5 mg·mL^−1^: C5 > F1, F2 and C3 **** and C5 > F3, C1, C2 and C4 ***.

We also evaluated the local toxicity of the formulations in RAW 264.7 macrophages. Similar to the results described above, the intergroup analysis showed that, in all concentrations, F1 decreased cell viability by approximately 50% when compared to control group formulations without SFN (*p* < 0.05). The other formulations (F2 and F3) also decreased cell viability when compared to control groups (*p* < 0.05), but this effect was less intense. For the intragroup analysis, the general finding was that the three formulations containing SFN (F1, F2, and F3) affected cell viability when compared to the HA formulation without SFN (C5) (*p* < 0.05) (Figure 2).

**Figure 3:**
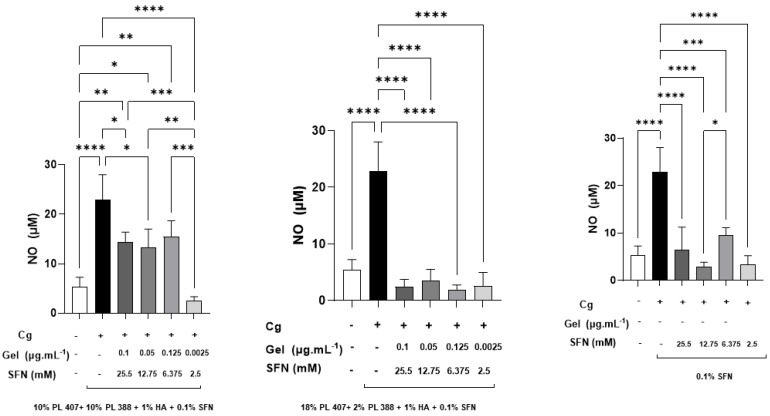
SFN formulations inhibit NO (Mean ± SD) production on RAW 264.7 macrophages after 24 h of treatment (F1, F2, and F3). One-way analysis of variance with Tukey post-hoc test: **** *p* < 0.0001; *** *p* < 0.001; ** *p* < 0.01; * *p* < 0.05. The intragroup analysis is represented in the figure with brackets. Intergroup analysis: Concentration 25.5; 12.75 and 6.375—F2 < F1 and F3 (*p* < 0.05).

Following the viability assessments, we collected supernatant cells from the cultures treated with SFN formulations (F1, F2, and F3) for nitric oxide determination. F1 at 2.5 mM was able to reduce NO production when compared to F2, F3, and carrageenan positive control group (*p* < 0.05). F2 presented the same effect in all concentrations when compared to the positive control group (*p* < 0.05). F3 also induced NO reduction in the same way. In addition, we found that there were significant differences between SFN concentrations of 12.75 mM and 6.375 mM SFN (*p* < 0.05). The intragroup analysis showed that at 25.5 mM, 12.75 mM, and 6.375 mM, F2 reduced NO production when compared to F1 (*p* < 0.05). The lowest SFN concentration (2.5 mM) did not produce significant differences when compared to other formulations (Figure 3).

The formulations used in our study were previously described by Monteiro de Nascimento et al. (2021) [1]. However, 3T3 and RAW 264.7 cells were first used in the present study. The former paper observed that, after treatment with SFN in hydrogels (PL407/PL338 + HA), SW2353 and M3T3 cells showed viability in the range of 102 to 116% and higher than 75%, respectively. In our study, the results were similar: 3T3 cell viability was 83 to 132%, and RAW 264.7 cell viability was 25 to 100%. Additionally, both studies showed that the cytotoxic effect was higher when PL and SFN concentrations were enhanced.

The in-vitro assay also showed that our formulations were able to reduce NO production. By itself, SFN reduces iNO expressions and inhibits NO production. These effects relate to its modulating effect on NF-κB and Nrf2 pathways [1,16]. In our study, we found that F2 reduced NO production more than F1 and F3. In this sense, it seems that the use of hydrogels enhances SFN’s anti-inflammatory activity.

**Figure 4:**
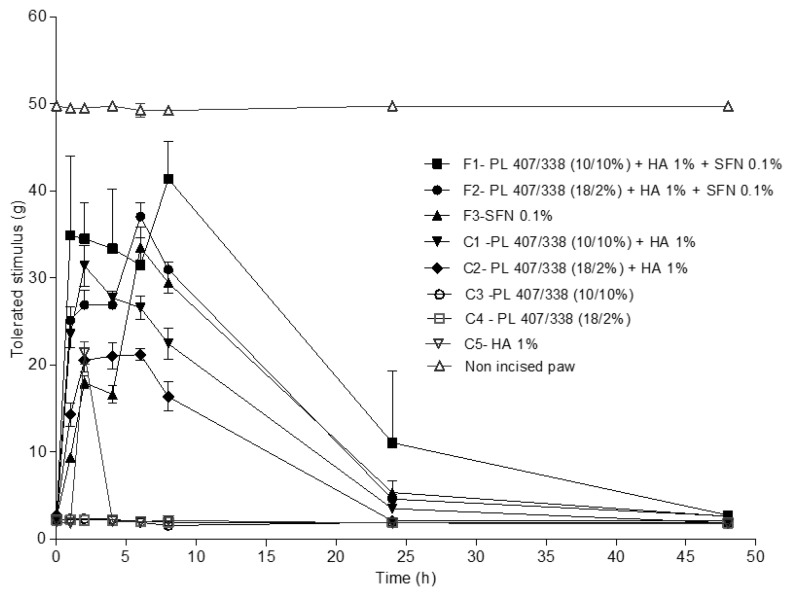
Maximum tolerated stimulus (mean ± SD) in grams after administration of SFN and control group formulations through intramuscular injections.

To demonstrate the effectiveness of these new formulations, we started by establishing that the in-vivo experimental model was able to produce mechanical hyperalgesia (Figure 4). There was a significant difference in animals’ basal levels of pain tolerance when comparing the non-incised and the incised paw. This held true for all periods of time up to 48 h (*p* < 0.001). The non-incised paw exhibited a maximum tolerated stimulus of approximately 50 g throughout the evaluation period. For the incised paw of animals in the control groups (PL without SFN and HA), this value was around 2–5 g (Figure 4). C3 and C4 did not present any significant differences during the evaluation period, which demonstrates that PL alone presents no analgesic effect after systemic administration.

**Figure 5:**
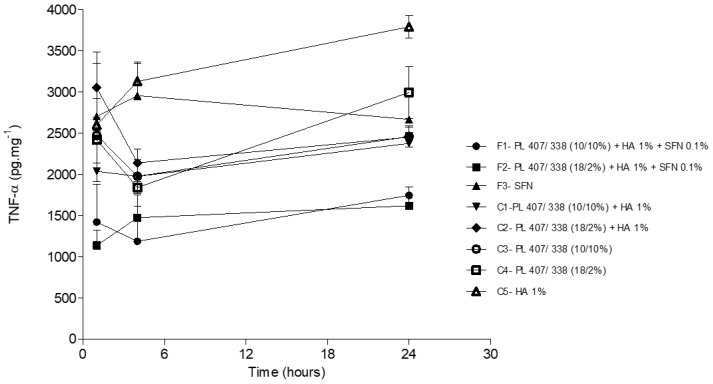
TNF-α levels in plantar tissue 1, 4, and 24 h after injection of SFN and control group formulations. Statistical analysis ANOVA/Tuckey-Kramer: F1 and F2 < F3 after 1 (*p* < 0.05), 4 (*p* < 0.01) and 24 h (*p* < 0.05). F1 and F2 < C5 after 1 (*p* < 0.05), 4 (*p* < 0.01) and 24 (*p* < 0.001).

F2 (PL 407/338 (18/2%) + HA 1% + SFN 0.1%) showed an increase in the tolerated stimuli over time (Figure 5) in almost all periods of evaluation. After 1, 2, and 4 h, F2 presented a higher mean value of tolerated stimulus when compared to the other formulations (*p* < 0.001). Six hours after the injections, F1, F2, and F3 all showed similar effects. Nevertheless, after 8 and 24 h, we once again observed an increase in the tolerated stimuli in animals treated with F2, when compared to groups that received other formulations (*p* < 0.001). This demonstrates the slow release of SFN when administered with these polymer combinations. After 48 h, none of the tested formulations presented analgesic effects. Table 1 shows these results, considering the tolerated stimuli over time.

To estimate the total analgesic effects of each formulation, we generated graphs of the tolerated stimulus versus time and calculated the areas under the curve (AUCs) through trapezoidal approximation, beginning at time zero and ending at 48 h [17]. When comparing AUCs, we observed a significant difference between F2 (678 ± 106) and all the other formulations, including those administered in the control groups (*p* < 0.05) (Figure 2). F1 (510 ± 36) presented higher AUC values when compared to all control groups (C1–410 ± 54; C2—295 ± 54; C3—44 ± 3; C4—48 ± 5; and C5—78 ± 9; *p* < 0.001). However, F3 (445 ± 58) presented a higher AUC value when compared to F1 (*p* < 0.05). Thus, our results indicate that SFN was able to reduce mechanical hyperalgesia and that this effect was more intense when the drug was administered using a hydrogel (F2).

Other studies have reached similar results, reporting SFN’s antinociceptive effect on different animal pain models. Fu et al. (2021) [18] used a cancer-induced bone pain model to establish the anti-hyperalgesic effects of SFN administered through intrathecal injections. Redondo et al. (2017) [19] likewise observed that SFN inhibited oxidative stress and inflammatory responses induced by peripheral inflammation. Finally, using a neuropathic pain model, Wang and Wang (2017) [20] reported that SFN administered through intraperitoneal injections presented both anti-nociceptive and anti-inflammatory actions in mice.

In our study, we used a specific surgery-related model developed in rats. Postoperative pain is a particular condition, as it is neither the result of an inflammatory process alone nor the result of an isolated injury to nerves. Pain behavior patterns observed in rodents subjected to surgical incisions indicate activation and sensitization of peripheral nociceptors and spinal dorsal horn neurons [21]. Evidence suggests that peripheral nociceptor sensitization and mechanical hyperalgesia are related to numerous targets such as NF-κB, TNF-α, IL-1β, substance P, IL-10, and iNOS, among others [13,22]. Thus, our study measures these substances to determine the antinociceptive effect of SFN.

**Figure 6:**
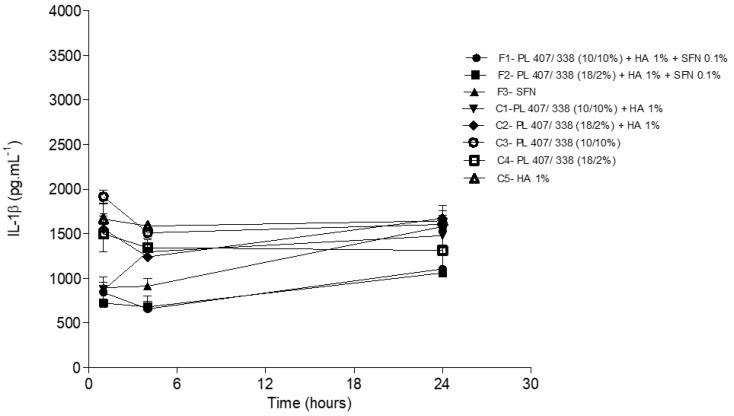
IL-1β levels in plantar tissue 1, 4, and 24 h after injection of SFN and control group formulations. Statistical analysis ANOVA/Tuckey-Kramer: F1, F2, and F3 < C5 after 1 (*p* < 0.05) and 4 h (*p* < 0.001).

Similarly to what was reported in the previously discussed studies [18,19,20,21,23], we observed that SFN’s antinociceptive effect was related to lower levels of TNF-α and IL-1β. These effects were more intense in formulations using hydrogels and are probably related to these systems’ ability to inhibit COX-2. The formulations’ ability to inhibit COX-2 was established in our group’s previous research using in-vitro experiments [1]. When compared to the control groups and isolated SFN (F3), SFN hydrogels (F1 and F2) reduced TNF-α levels 1, 4, and 24 h after the intramuscular injections (*p* < 0.05) (Figure 5). Concerning IL-1β levels, we observed significant differences between F1, F2, and F3 when compared to C5 after 1 and 4 h (*p* < 0.05) (Figure 6). Twenty-four hours after the injections were administered, there were no significant differences in IL-1β levels among the groups

**Figure 7:**
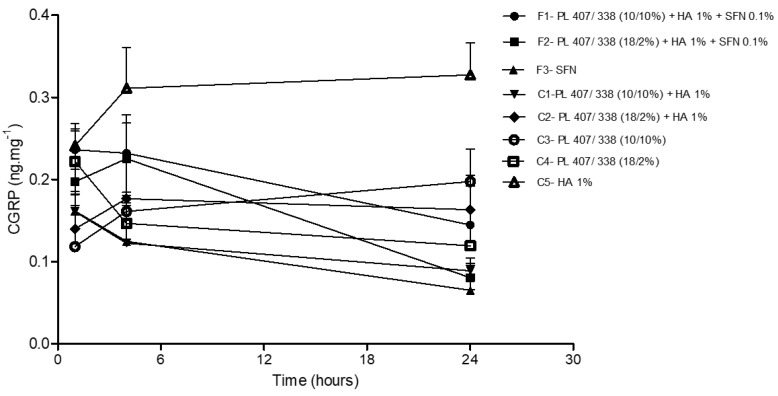
CGRP levels in plantar tissue 1, 4, and 24 h after SFN formulations and control group injections. Statistical analysis ANOVA/Tuckey-Kramer: F1, F2 and F3 < F8 after 24 h (*p* < 0.05).

We also assessed substance P and CGRP levels in the incised paw tissue. After 1 and 4 h, CGRP levels were similar across all formulations and control groups (*p* > 0.05). Twenty-four hours after the injections were administered, F1, F2, and F3 reduced CGRP levels when compared to C5 (*p* < 0.05) (Figure 7). Substance P levels at all-time points for all formulations were below the limit of quantification determined by the ELISA kit. Therefore, these values were not used in the statistical analysis.

**Figure 8:**
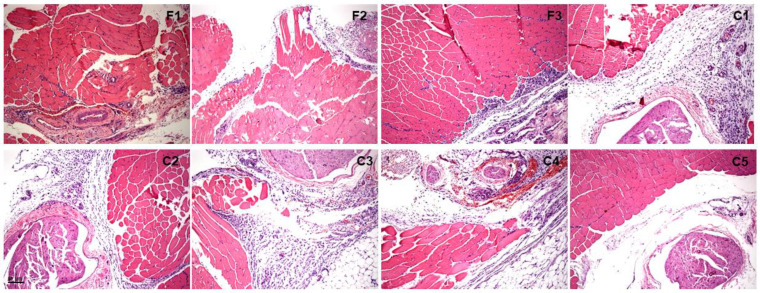
Histological analysis of the sciatic nerve region in rats 2 days after administration of treatment and controls. Scale bar: 50 µm.

**Figure 9:**
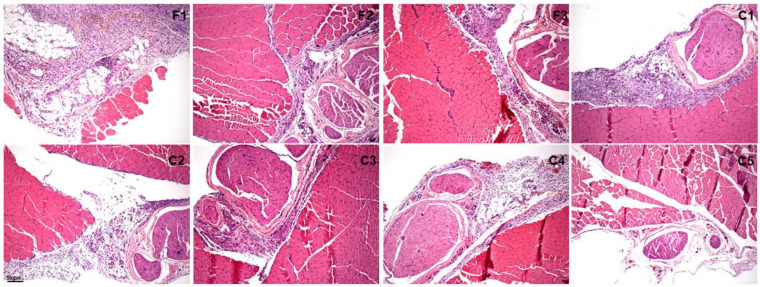
Histological analysis of the sciatic nerve region in rats 7 days after administration of treatment and controls. Scale bar: 50 µm.

Figure 8 and Figure 9 display cross-sections of the injection site (intramuscular administration) and surrounding soft tissues 2 and 7 days after injection of the tested formulations and their respective control groups. Two days after administration, there were no significant differences among all tested formulations and the injections received by the control groups (*p* < 0.05, Figure 9 and Table 2). However, C3 induced higher inflammatory reaction scores than F1, F2, F3, and C5 (*p* < 0.05). After 7 days, C5 promoted lower inflammatory reaction scores than F1, C1, and C3 (*p* < 0.05).

## 3. Conclusions

The results indicate that the presence of PL and HA enhances SFN’s effect on postoperative pain relief. Combining PL 407/338 (18/2%) and HA 1% with SFN 0.1% (F2) extended SFN’s bioavailability, provoking longer and more intense analgesic effects on treated rats. The improved effects of SFN administered in this combination were additionally demonstrated by the lower levels of TNF-α, IL-1β, and CGRP. Finally, in-vitro tests and histopathological analysis of the injection sites corroborated this formulation’s safety. This suggests that SFN hydrogels could be a promising strategy for controlling inflammatory pain after surgery. Effective management of postoperative pain enhances patient functionality and quality of life while also reducing morbidity, recovery time, opioid use, and healthcare costs. Therefore, SFN hydrogels might serve as a non-opioid alternative for controlling postoperative pain, addressing the inflammatory response without the side effects of NSAIDs or the risks associated with opioids.

## 4. Materials and Methods

### 4.1. Preparation of Hydrogels

We prepared the formulations to be applied in the study as previously described in Monteiro do Nascimento et al. (2021) [1]. The poloxamer (PL) (Sigma-Aldrich Chem. Co., St. Louis, CA, USA) solutions (20%) (m/v), composed of binary systems of PL407-PL338 (10–10% or 18–2%), were dissolved in cold water under stirring (100 rpm) at 4 °C until a transparent solution was formed. Hyaluronic acid (HA) (MW 1500–1750 kDa, Sigma-Aldrich Chem. Co., St. Louis, CA, USA) (1%) and sulforaphane (0.1% SFN, Toronto Research Chemicals, Toronto, ON, Canada) were then incorporated into the PL solutions simultaneously.

The investigation selected formulations based on previously reported rheological properties. This selection resulted in the use of the following formulations: F1-PL 407/338 (10/10%) + HA 1% + SFN 0.1%; F2-PL 407/338 (18/2%) + HA 1% + SFN 0.1%; F3-SFN 0.1%. We also established control groups, which received one of five formulations: C1-PL 407/338 (10/10%) + HA 1%; C2-PL 407/338 (18/2%) + HA 1%; C3-PL 407/338 (10/10%); C4-PL 407/338 (18/2%); C5-HA 1%.

### 4.2. In-Vitro Cell Viability Assays

For the in-vitro assays, this study used Balb/c 3T3 and RAW 264.7 cells. We seeded Balb/c 3T3 cells in 96-wells plates (2 × 10^4^ cells/well) for 48 h, under a humidified atmosphere (37 °C and 5% CO_2_), using Dulbecco’s Modified Eagle Medium (DMEM) (Gibco Laboratories, Grand Island, NY, USA) supplemented with 10% (*v*/*v*) fetal bovine serum (Sigma Chemical Co., St. Louis, MO, USA) (pH 7.2–7.4) and 100 μg/mL of penicillin/streptomycin (GE Healthcare, Chicago, IL, USA). Next, we homogenized predetermined amounts of SFN formulations and control group formulations in DMEM using a vortex for 5 min. We then used the resulting solutions to treat the cells for 24 h.

To evaluate cell viability (3T3), we added a Methylthiazolyldiphenyl-Tetrazolium bromide (MTT) solution (100 µL, at 5 mg/mL in solution in phosphate buffered saline) to the plates and incubated it for 4 h. After this period, the MTT solution was removed, and Dimethyl sulfoxide (DMSO) (50 μL) was added to the plates for 10 min. We measured absorbance at 570 nm. The control group that received a non-toxic formulation was treated with DMEM under the same conditions used for the plates that received SFN formulations [12].

Regarding RAW 264.7, our study grew macrophages in RPM1 1640 medium (Sigma Chemical Co., St. Louis, MO, USA) supplemented with 10% fetal bovine serum (Sigma Chemical Co., St. Louis, MO, USA) and 1% penicillin/streptomycin (GE Healthcare, Chicago, IL, USA). We then plated the cultures at a density of 1 × 10^6^ cells per well, in a 96-well plate, and assessed cell viability with tripan blue assays. For this, we primed cells with carrageenan (300 µg·mL^−1^) [24] and treated them with predetermined amounts of SFN and control group formulations. After 24 h of stimulation with carrageenan, we collected supernatant cells for Nitric Oxide (NO) determinations (Griess Reagen Kit, Invitrogen^®^, Eugene, OR, USA).

For both assays, this study used SFN and control group formulations that had the same serial dilution. The initial concentration for SFN formulations was 56 mM, and cells were treated in a concentration interval from 2.5 to 25.5 mM. The initial concentration of PL and HA in control groups was 0.2 µg·mL^−1^ and 10 mg·mL^−1^, respectively. The PL concentrations ranged from 0.1 to 0.0125 µg·mL^−1^; while HA concentrations ranged from 5 to 0.625 mg·mL^−1^. We performed intra- and intergroup comparisons to evaluate differences between the four tested concentrations and between the total of eight groups, respectively.

### 4.3. Postoperative Pain Model in Rats

**Figure 10:**
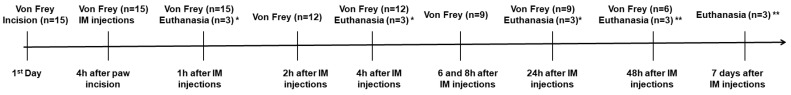
Flowchart of experiments for induction and treatment of postoperative pain in rats’ right hind paws with SFN and control group formulations. For comparison, the animals’ left hind paws were tested in the same periods. The flowchart presents only one experimental group, but the same procedure was adopted in all 8 experimental groups. * Scar tissue removal for ELISA (TNF-α, IL-1β, Substance P, and CGRP). ** Injection site tissue removal for histopathological analysis.

The investigation used 120 male Wistar rats (300–350 g). Animals were housed in groups of 5 per cage and received water and food *ad libitum* with a 12:12 h light-dark cycle, at 23 ± 2 °C. The experimenters handled them 7 days prior to the research procedures to promote acclimatization with the test site, apparatus, and the researchers themselves. The animals were then divided into groups according to the flowchart represented in Figure 10.

Prior to the study’s execution, we submitted the experimental protocol to the Animal Experimentation Ethics Committee of the São Leopoldo Mandic Research Institute and Center (Protocol n. 2019/043), where it was subsequently approved. Our research group designed it to minimize animals’ stress and pain and to use the smallest possible number of animals.

Regarding experimental procedures, we first placed animals in clear plastic cages with elevated wire mesh floors and recorded their basal withdrawal responses to the von Frey anesthesiometer (Ugo Basile, Varese, Italy). The withdrawal response consists of the animals’ elevation of their paw when experimenters probed the plantar region with a growing pressure, measured in grams. Next, we induced anesthesia with a sevoflurane-soaked gauze at the bottom of a 50 mL Falcon^®^ tube placed over the animals’ muzzles for 10 to 15 s [13,17,25].

We then made a 1-cm-long incision on the animals’ right hind paws, cutting through skin, fascia, and muscle at the most lateral plantar region, as described in Wang et al.’s model [26]. Experimenters closed the wound with a 5–0 nylon suture. 4 h after the incision, the animals were divided into eight groups (n = 15) and received an intramuscular injection in the posterior face of the right thigh (0.2 mL). These injections contained one of the tested formulations or one of the control group formulations described in item 2.1.

After 1, 2, 4, 6, 8, and 24 h, our team assessed the mechanical hypersensitivity in both the right (with the incision) and the left (untreated) paws [13,27,28]. We considered that the absence of a withdrawal response when probing animals’ paws with a stimulus equal to or more intense than the basal levels registered in the first step represented successful analgesia. This criterion was also used to determine the duration of SFN formulations’ effects [25,29,30]. To estimate the total analgesic effect of each individual formulation, we generated graphs representing tolerated stimulus versus time and calculated the area under the curve (AUC) through trapezoidal approximation, beginning at time zero and ending at the last time in which the tolerated stimulus was above the basal threshold [13,17].

At intervals of 1, 4, and 24 h after treatment using the SFN and control group formulations, three animals in each group were euthanized after the measurement of their mechanical hypersensitivity. This was performed to assess the scar tissue on their right hind paws with ELISA. Experimenters removed plantar tissue samples from the area, froze them at −80 °C, and assessed their levels of TNF-α, IL-1β, substance P, and CGRP. This was performed by following the procedures prescribed in the guidelines and the protocols of the commercial ELISA kit [13]. This design allowed us to further monitor the analgesia’s duration and determine the concentration of cytokines in affected tissues.

As a final step, our team euthanized the remaining animals 24 h and 7 days after they received their injections (n = 3). We then removed the soft tissue surrounding the injection site and prepared samples to obtain five cross-sections (5 μm thick, 40 μm deep) stained with hematoxylin and eosin. These cross-sections were submitted to qualitative analysis. This procedure was performed by a blinded subject and used a specific scoring system [17]. The analyzed region was the site of the injection, which included the soft tissue surrounding the sciatic nerve. We also photographed the cross sections with a photomicroscope. The score of local tissue inflammation was defined based on the following descriptions: (1) less than 25% of the total area presented no infiltrate, injury, or necrosis (mild inflammation); (2) between 25% and 50% of the evaluated area presented inflammatory infiltrate, injury, or necrosis (moderate inflammation); (3) more than 50% of the area was injured or presented necrosis areas (severe inflammation) [17].

### 4.4. Statistical Analysis

Graph PadPrism 6.0 (GraphPad Software, Inc., La Jolla, CA, USA) was used for all statistical calculations. We submitted the results from the postoperative pain model to a two-way analysis of variance (ANOVA), followed by the Bonferroni post-hoc test. The sample size was calculated using the findings from our previous work [13,17] and was based on the equation for a finite population [31].

## Figures and Tables

**Table 1:** Mean (±SD) tolerated stimuli in grams after formulations and control group administration via the intramuscular route.

	1 H	2 H	4 H	6 H	8 H	24 H
F1-PL 407/338 (10/10%) + HA 1% + SFN 0.1%	25.12 ± 4.69 a ***. b ***. d ***. e ***. f ***. g ***	26. 91 ± 5.09 a **. b ***. d *. e ***. f ***	26.91 ± 3.93 a **. b ***. d *. e ***. f ***. g ***	37.59 ± 4.98 c *. d ***. e ***. f ***. g ***	30.97 ± 2.60 a ***. c ***. d ***. e ***. f ***. g ***	4.61 ± 1.62 a **
F2-PL 407/338 (18/2%) + HA 1% + SFN 0.1%	34.89 ± 9.09 h ***. i ***. j ***. k ***. l ***. m ***	34.51 ± 4.09 h ***. j ***. k ***. l ***. m ***.	33.36 ± 6.87 h ***. i *. j ***. k ***. l ***. m ***	31.85 ± 3.92 j **. k ***. l ***. m ***	41.37 ± 4.25 h ***. i ***. j ***. k ***. l ***. m ***	11.07 ± 8.20 h *. i ***. j ***. k ***. l ***. m ***
F3-SFN 0.1%.	9.39 ± 1.17 n ***. p *. q *. r **	17. 98 ± 2.17 n ***. p ***. q ***	16.63 ±2.89 n ***. p ***. q ***. r ***	32.61 ± 6.30 o ***. p ***. q ***. r ***	29.44 ± 3.63 n **. o ***. p ***. q ***. r ***	5.34 ± 3.98
C1-PL 407/338 (10/10%) + HA 1%	23.54 ± 4.76 s ***. t ***. u ***. v ***	31. 38 ± 7.03 s ***. t ***. u ***. v ***	24.72 ± 2.38 s **. t ***. u ***. v ***	29.84 ± 6.20 s *. t ***. u ***. v ***	22.45 ± 5.30 s **. t ***. u ***. v ***	3.49 ± 1.11
C2-PL 407/338 (18/2%) + HA 1%	14.32 ± 3.96 w ***. x ***. y ***	20.54 ± 4.20 w ***. x ***.	21.03 ± 4.44 w ***. x ***. y ***	22.65 ± 4.55 x ***. y ***. z ***	16.37 ± 5.00 x ***. y ***. z ***	2.096 ± 0.32
C3-PL 407/338 (10/10%)	2.32 ± 0.39	2.35 ± 0.34 aa ***	2.27 ± 0.38	2.21 ± 0.43	1.56 ± 0.32	1.91 ± 0.30
C4-PL 407 − 18% + PL 188 − 2%	2.20 ± 0.54	2.18 ± 0.31 bb ***	2.18 ± 0.31	2.11 ± 045	1.98 ± 0.43	2.01 ± 0.35
C5-HA 1%	1.81 ± 0.39	21.39 ± 3.95	2.06 ± 0.33	2.04 ± 0.46	2.13 ± 0.38	1.85 ± 0.40

Note Statistical Analysis [ANOVA/Tukey- Kramer- * (*p* < 0.05); ** (*p* < 0.01), *** (*p* < 0.001)]: a F1 vs. F2; b F1 vs. F3; c F1 vs. C1; d F1 vs. C2; e F1 vs. C3; f F1 vs. C4; g F1 vs. C5; h F2 vs. F3; i F2 vs. C1; j F2 vs. C2; k F2 vs. C3; l F2 vs. C4; m F2 vs. C5; n F3 vs. C1; o F3 vs. C2; p F3 vs. C3; q F3 vs. C4; r F3 vs. C5; s C1 vs. C2; t C1 vs. C3; u C1 vs. C4; v C1 vs. C5; w C2 vs. C3; x C2 vs. C4; y C2 vs. C5; z C3 vs. C4; aa C3 vs. C5; bb C4 vs.C5.

**Table 2:** Median (upper and lower limits) inflammatory reaction scores 2 and 7 days after intramuscular administration of tested and control group formulations.

Formulations	2 Days	7 Days
F1-PL 407/338 (10/10%) + HA 1% + SFN 0.1%	1 (1–3)	2 (2–3)
F2-PL 407/338 (18/2%) + HA 1% + SFN 0.1%	1 (1–2)	2 (1–3)
F3-SFN 0.1%.	1 (1–2)	2 (1–2)
C1-PL 407/338 (10/10%) + HA 1%	2 (1–3)	3 (2–3)
C2-PL 407/338 (18/2%) + HA 1%	2 (1–3)	2 (1–2)
C3-PL 407/338 (10/10%)	3 (2–3) a *, b *, c *, d *	2 (2–3)
C4-PL 407 − 18% + PL 188 − 2%	2 (2–3)	2 (2–3)
C5-HA 1%	1 (1–2)	2 (1–2) e **, f ***, g ***

Statistical analysis Dunn: * *p* < 0.05; ** *p* < 0.01; *** *p* < 0.001. a—F1 vs. C4; b—F2 vs. C3; c—F3 vs. C3; d—C5 vs. C3; e—F1 vs. C5; f—C1 vs. C5; g—C3 vs. C5.

## Data Availability

All data and materials are available on request from the corresponding author. The data are not publicly available due to ongoing researches using a part of the data.
